# Metronomic doses and drug schematic combination response tested within chambered coverslips for the treatment of breast cancer cells (JIMT-1)

**DOI:** 10.1371/journal.pone.0274911

**Published:** 2022-09-29

**Authors:** Gustavo Rosero, Gisela Pattarone, Ana Peñaherera, Julia Pilz, Joschka Bödecker, Maximiliano Perez, Roland Mertelsmann, Betiana Lerner, Marie Follo

**Affiliations:** 1 National Technological University (UTN) IREN Center, Buenos Aires, Argentina; 2 Faculty of Medicine, Department of Medicine I, Medical Center–University of Freiburg, University of Freiburg, Freiburg, Germany; 3 Faculty of Engineering, Neurorobotics Lab, Computer Science Department, University of Freiburg, Georges-Köhler-Allee, Freiburg, Germany; 4 Faculty of Engineering, University of Buenos Aires (UBA), Institute of Engineering Biomedical, Buenos Aires, Argentina; 5 Department of Electrical and Computer Engineering, Florida International University, Miami, FL, United States of America; 6 Collaborative Research Institute Intelligent Oncology, Hermann-Herder-Straße, Freiburg im Breisgau, Germany; UNITED STATES

## Abstract

Low-dose metronomic (LDM) chemotherapy is an alternative to conventional chemotherapy and is the most frequently used approach in low dose chemotherapy regimens. The selection of patients, drug dosages, and dosing intervals in LDM is empirical. In this study, we systematically examined the schedule-dependent interaction of drugs on a breast cancer cell line (BCC) cultured in chambered coverslips. The LDM studies were combined with cell staining in order to better characterize different cell states and cell death modes, including caspase-dependent apoptosis, caspase-independent cell death and autophagy-dependent cell death. Microscope images were examined using the Fiji Trainable Weka Segmentation plugin to analyse cell area in 7500 images showing different modes of cell death. Paclitaxel combined with LDM chemotherapy demonstrated a reduction in the area covered by live cells. In contrast, there was an induction of high levels of cell death due to caspase-dependent apoptosis.

## Introduction

Conventional *in vitro* 2D assays have been widely used to evaluate the role of chemoattractants on cancer cell migration [1,2]. On the one hand, *in vitro* culture models offer the advantage of having well-established protocols. However, they do not allow for variation of nutrient and waste concentrations [3]. Therefore, in this study we used chambered coverslips to cultivate breast cancer cells, in order to apply metronomic cancer treatments and to assess cell death modes, while maintaining the benefit of low reagent volumes. Coverslips have been specifically designed for cell culture, immunofluorescence and high-end microscopic analysis. Furthermore, each coverslip has 8 chambers which enhances reproducibility. In fact, Jorge-Peñas *et al*., [4] utilized chambered coverslips to measure cell-induced deformation of the surrounding matrix. In fact, staining cells inside chambered coverslips allows high-resolution microscopy and is a powerful tool to detect intracellular changes through live cell imaging. It enables the determination of changes such as distinguishing live from dead cells, lysed or senescent cells and other dynamic changes in cell state [5–7]. The characteristics of the μ-Slide 8-well chambered polymer bottom coverslips are suitable for cancer cell research because they enable cultivation, solution exchange [8] and high-resolution and fluorescence microscopy of living cells, even over extended time periods [9]. This allowed us to track changes in real time and to acquire enough images to train the WEKA segmentation algorithm, which was used to quantify the area occupied by cells and to classify it according to cellular fluorescence.

Discovering ways to fight or block malignant cell growth has been a driving force in cancer biology research over the past four decades [10]. For this reason, being able to develop new approaches for cell analysis will be a useful tool in this area.

Human breast cancer (BC) is the most common malignancy in women [11]. This type of cancer is comprised of a phenotypically diverse population of breast cancer cells (BCC) [12]. Paclitaxel has been demonstrated to have initial activity in BC [13] and if combined with doxorubicin, is the most active agent known to work against BC when using different schedules [14]. Low-dose metronomic (LDM) chemotherapy is a novel use of chemotherapy, and is defined as using conventional low doses without prolonged drug-free periods [15]. This approach is clinically relevant because *in vitro* LDM induces a synergistic cytotoxic effect in triple negative breast cancer cell lines, and has been applied in clinical phase I/II trials of advanced breast cancer patients [16,17]. The JIMT-1 cell line behaves like a triple negative cell line because it lacks the estrogen and progesterone receptors and is insensitive to trastuzumab. Therefore, this study represents an experimental setup which can validate protocols being used in current clinical trials for breast cancer treatments.

Anticancer medications can incite different modes of cell death, including caspase- dependent apoptosis, caspase-independent cell death, reproductive cell death (cell senescence), or cell death due to autophagy [18]. These non-standard pathways show the unexpected intricacy of molecular crosstalk within the cell death pathways, reflected in a diversity of phenotypes which have yet to be characterized [19].

Considering the benefits of chambered coverslips with the advantages of using low numbers of cells and reagents, the present study combines biology, computer science and microfluidics to focus on the characterization of biological behavior of breast cancer cells (JIMT-1). The study uses different stainings to evaluate cell viability (CV), caspase-dependent programmed apoptosis (AP), autophagy (AG), and cell death (CD), examining the samples before and after the introduction of two different types of drug treatments applied *in vitro* to the breast cancer cells in the chambers. Live-cell image acquisition enabled the characterization of different cell death modes by applying machine learning to analyze a large number of images.

## Materials and methods

### Cell culture

JIMT-1 cells ATCC 589 (DSMZ) were cultured in complete DMEM medium (Gibco), supplemented with heat-inactivated fetal calf serum (FBS) 10% (w/v) (Gibco), L-glutamine 2mmol·L^−1^ (Gibco), penicillin 100 units·mL^-1^, streptomycin 100 μg·mL (Gibco) at 37°C in an incubator with 5% CO_2_. For microscopy assays, 20.000 cells per well were cultured in 8-well μ-Slide chambered polymer bottom coverslips (cat. no. 808126, Ibidi GmbH).

### Cell death modes and characterization

**Fig 1:**
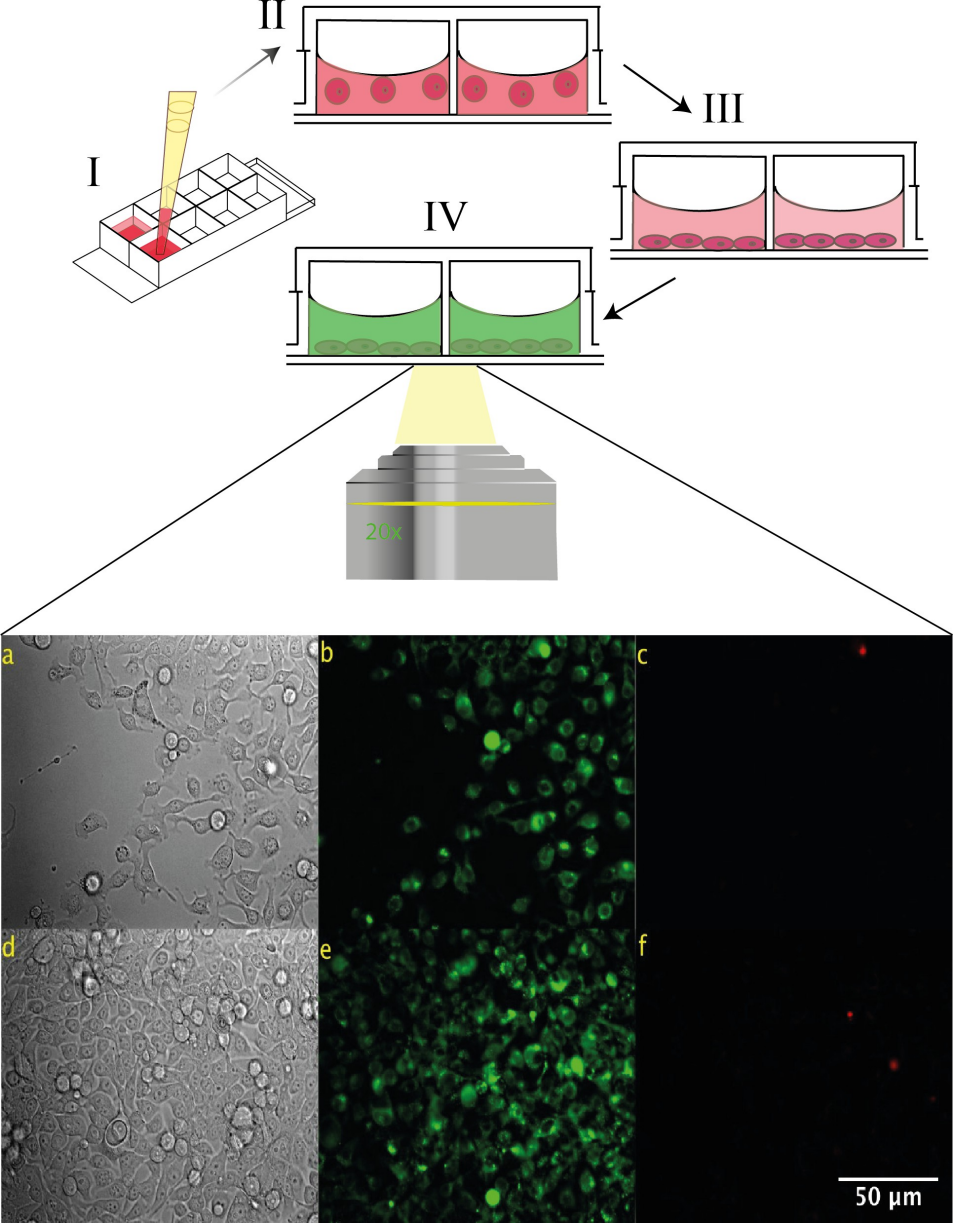
Representative microscopy images of breast cancer cells after three days in the microdevice. (a,d) Brightfield images of the culture. (b,e) Fluorescence images of cells after treatment with the autophagy kit, which preferentially labels autophagous vacuoles, shown here in green (c,f) Fluorescence images after treatment of the cells with CD. Dead cells are shown in red.

The Live/Dead Cell Imaging Kit (Sigma), the Autophagy Cell Imaging Kit (CYTO-ID), the Caspase-3 and -7 Cell Imaging Kit (Invitrogen), and Propidium iodide to detect cell death (CD) (Sigma-Aldrich) were used to identify and distinguish the different modes of cell death. For the Live/Dead (CV) assay, viable cells were stained green, while dead cells were stained red [20]. Autophagous cells in the autophagy assay (AG) were stained green (Fig 1), and negative and positive controls were performed as recommended by the manufacturer`s instructions (Enzo ENZ-51031- K200) [21]. In the caspase-3 and -7 assay (AP), apoptotic cells were stained green [22]. In the CD assay, dead cells were stained red. The cells were loaded into the chambered coverslip, as described previously, and CD, CV, AG, AP were evaluated on the first, third, fourth and fifth days after treatment. We used phosphate buffered saline (PBS, HyClone) to wash the culture chambers in the models for 3 min. Cells were then incubated with the appropriate stain for 30 min at 37°C, respectively. After this, the staining buffer was then removed and PBS was placed in the well for 5 min to wash out the staining reagent. Before examining the culture chambers under a fluorescent microscope, the chambered coverslip was filled with fresh DMEM media. Finally, cell viability was measured by assessing the percentage of fluorescent cells in the cell cultures.

### Combined treatment of cells with Paclitaxel and doxorubicin and examination of drug effects

**Fig 2:**
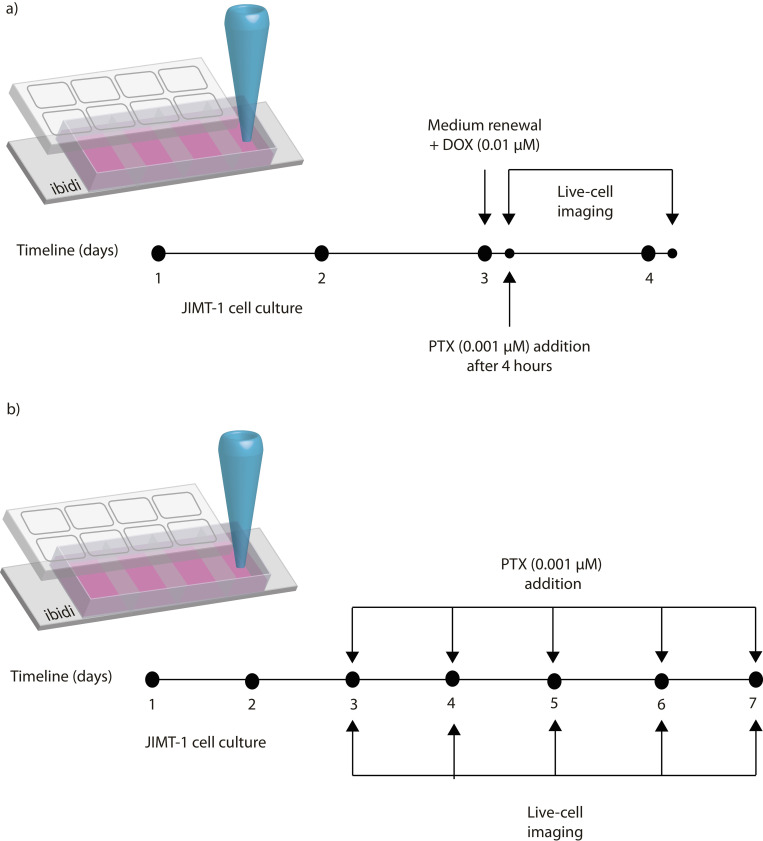
Timeline of applied pharmacological strategies. a) Combined DOX and PTX treatment DOX (0.01 μM) and 4 hours later PTX (0.001 μM). b) Metronomic PTX treatment (0.001 μM) for 5 days.

Paclitaxel and doxorubicin were added to the cells to test their effect, both alone and in combination (Sigma Aldrich) [23]. As illustrated in Fig 2A, JIMT-1 cells were plated on the chambered coverslips and were grown for two days. After 24 hours, the medium was replaced with fresh culture medium mixed with doxorubicin at a concentration of 0.01 μM. After four hours in doxorubicin, paclitaxel was added to the medium at a final concentration of 0.001 μM. The cells were cultured for a further 24 hours. Live cell imaging and biological characterization of the cells were carried out using the different stainings as described above. Characterization and cell death mode analyses were performed in order to evaluate the treatment efficiency. The first stage was after four hours of doxorubicin exposure and the second stage after doxorubicin and paclitaxel.

### Metronomic Paclitaxel treatment and evaluation of drug effects

For the metronomic Paclitaxel tests, the effects of paclitaxel when given at different time points were studied [15]. JIMT-1cells were first plated onto the chambered coverslips as described in Fig 2B. After 24 hours, the medium was replaced with fresh culture medium containing paclitaxel at a concentration of 0.001 μM. The medium containing 0.001 μM paclitaxel was then replaced every 24 hours for the next five days. Biological characterizations using the different stainings were performed as described above.

### Image processing and quantification

Brightfield and fluorescent images were obtained using an automated fluorescence microscope (Olympus ScanR High Content Screening Station) using the 20x UPLSAPO NA 0.75 objective. Cell images were classified according to cell viability (CV), programmed apoptosis (AP), autophagy (AG), or cell death (CD). In total, 7500 images were analyzed using the Fiji program (v.2.0.0) and the Trainable Weka Segmentation plugin (v.3.2.33): The Weka plugin within ImageJ is an open-source platform for biological-image analyses that combines a collection of machine learning algorithms with a set of selected image features to produce pixel-based segmentations [24,25]. All of the experiments were performed in triplicate and the data are presented as the mean ± standard deviation (SD). A one-way analysis of variance (ANOVA) and Student’s t-test were used for comparisons of each group. P-values less than 0.05 were considered statistically significant and are indicated with asterisks.

## Results

### Tracking the growth of breast cancer cells within the cell culture chambers

Evaluation of the different schematics and the efficacy of therapeutic drugs is important. Chambered coverslips were used to track and evaluate the development of breast cancer cells over a period of five days. To better understand the development and characterization of cells within the coverslip chambers, the percentages of live, dead, autophagous and apoptotic cells were analyzed over a period of five days.

**Fig 3:**
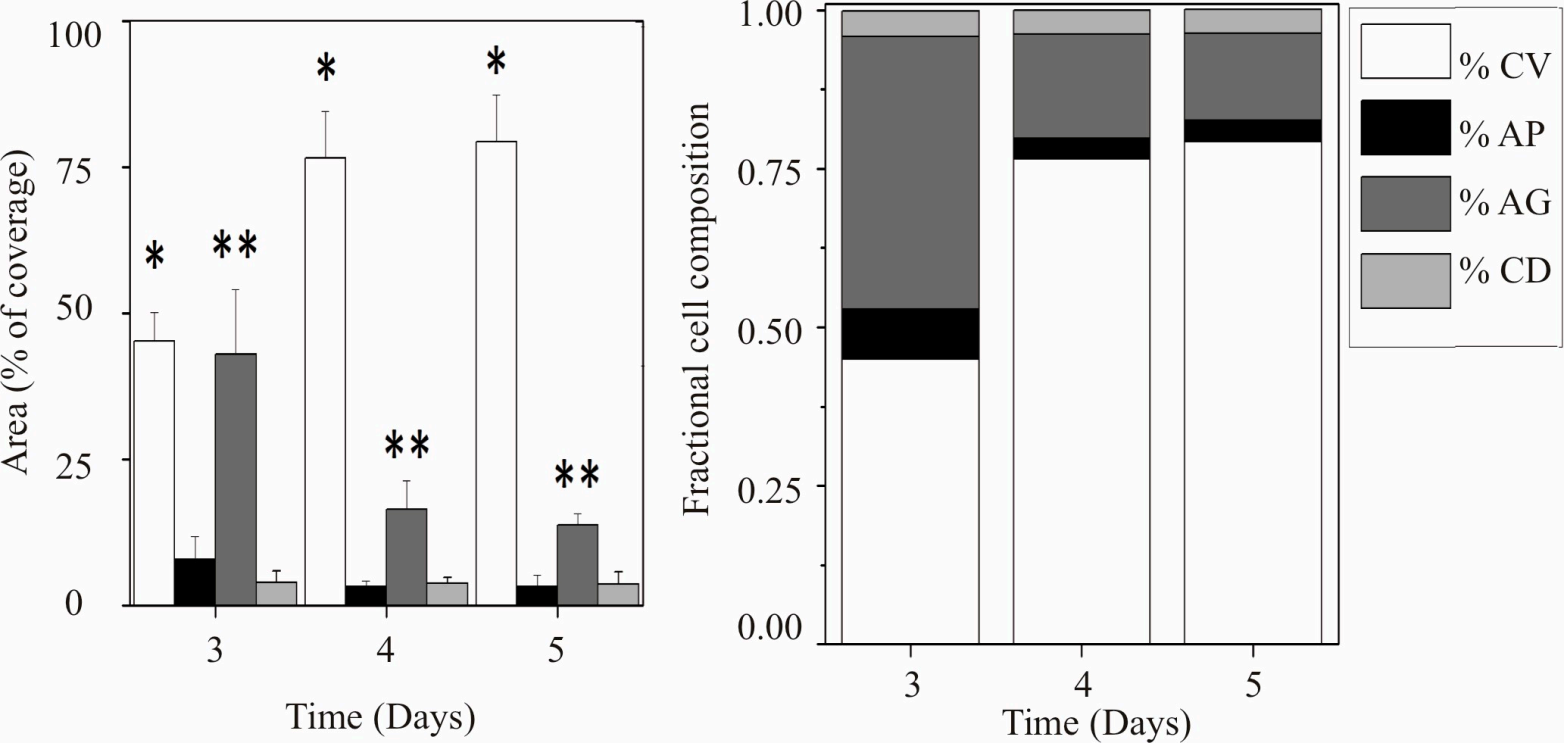
Cell distribution after characterization with stainings for 5 days. Right, stacked bar graph illustrating cell fraction composition per day over the five-day period. Left, the surface area covered by live cells (CV) increased while the levels of the autophagy process decreased (AG) significantly. (*) and (**) indicate significant difference between days among the subgroups (p < 0.05).

The results of growing cells under these different conditions can be observed in Fig 3.

Significant differences in the percentages of CV and AG were observed over the five-day time period. The percentage of live cells increased over the five-day period (Fig 3 left). In contrast, cells in autophagy were at a high percentage of total cell area on the third day and had decreased by the fifth day. Notably, there is growth of the cells over the five-day period, detectable when using the marker for live cells. The chambered coverslips were able to provide homogeneous conditions for keeping the cells viable in culture over this time period [26].

### Effect of Paclitaxel and doxorubicin drug schematics and the evaluation of CV, AG, CD and AP

**Fig 4:**
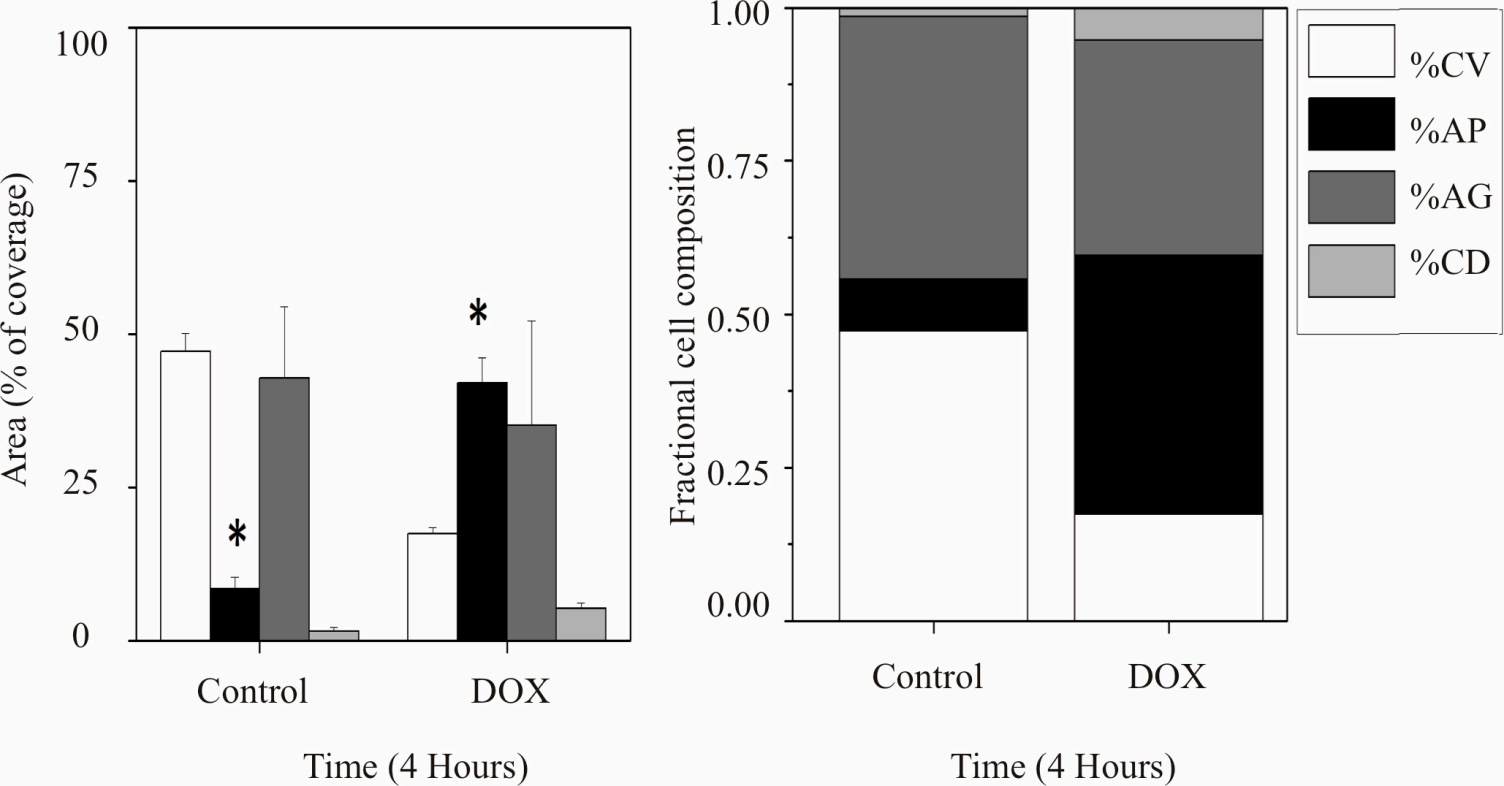
Doxorubicin effect on cells after four hours. Left, the apoptosis process (**AP**) increased significantly with respect to the control. The area covered by viable cells (**CV**) showed a decrease which was not significant in comparison to the control. Right stacked bar graph shows the value of the results in cell fraction composition. (*) indicates a significant difference between days among subgroups (p < 0.05).

There has been considerable interest in recent years in synergistic chemotherapeutic agents for the treatment of breast cancer [18,27]. Previous clinical trials have shown that there are benefits in combining DOX and PTX while treating breast cancer patients. However, side effects such as neutropenia and congestive heart failure have limited the dose which can be administered to patients [28–30]. To analyze the effect of drugs on cells using the chambered coverslips, it is appropriate to keep the cell culture growing under homogeneous conditions [31]. First, the effect of DOX was studied, and the analysis is described in Fig 4. Samples were incubated for four hours with DOX at a concentration of 0.01 μM. The level of apoptosis in the cells was estimated by quantifying the stained area relative to the entire cell population using the AP assay described above. Significant differences in apoptotic activity with respect to the control cells were found after four hours of exposure to DOX. The effect of DOX in the first four hours reduced the percentage in the area of living cells and produced an increase of apoptotic cells.

**Fig 5:**
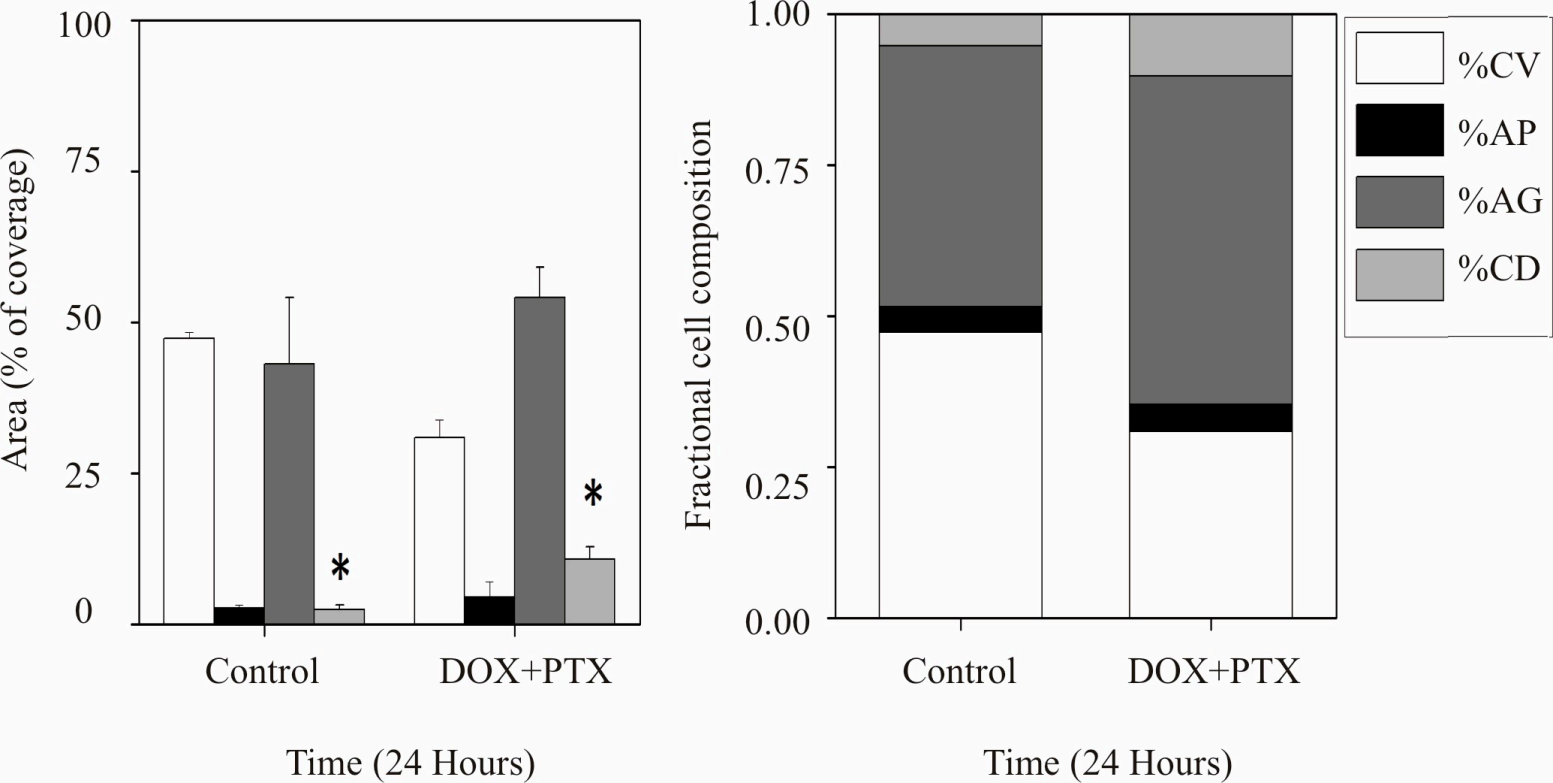
Doxorubicin combined with Paclitaxel. Bar graph denotes the effect of PTX after 24 hours on cells death modes. Propidium staining (**CD**) increased significantly with respect to the control. The area covered by live cells (**CV**) showed a decrease which was not significant in comparison to the control. Right stacked bar graph shows the value of the results in cell fraction composition after 4-hour exposure to doxorubicin and a combination of paclitaxel and doxorubicin per day. (*) Indicates significant difference with days among subgroups (p < 0.05).

The results obtained from the image analysis can be seen in Fig 5. No significant differences were seen in CV, AP and AG. However, the area stained for CD showed a slight but significant increase compared to the controls.

PTX combined with DOX is widely reported to have a deeply synergistic inhibitory effect on the growth of several breast cancer cell lines [32,33]. Our results (Fig 5) did not show further increases in the percentages of **AP** or in the inhibition of growth. This may be due to the fact that higher concentrations of PTX can actually have the opposite effect on cell growth, as has been previously reported [34]. Numerous factors contribute to paclitaxel resistance within a given cell population, and these factors were found to be highly variable. Therefore, the exact mechanism of action remains unclear [34,35].

### Effect of metronomic treatment with Paclitaxel

**Fig 6:**
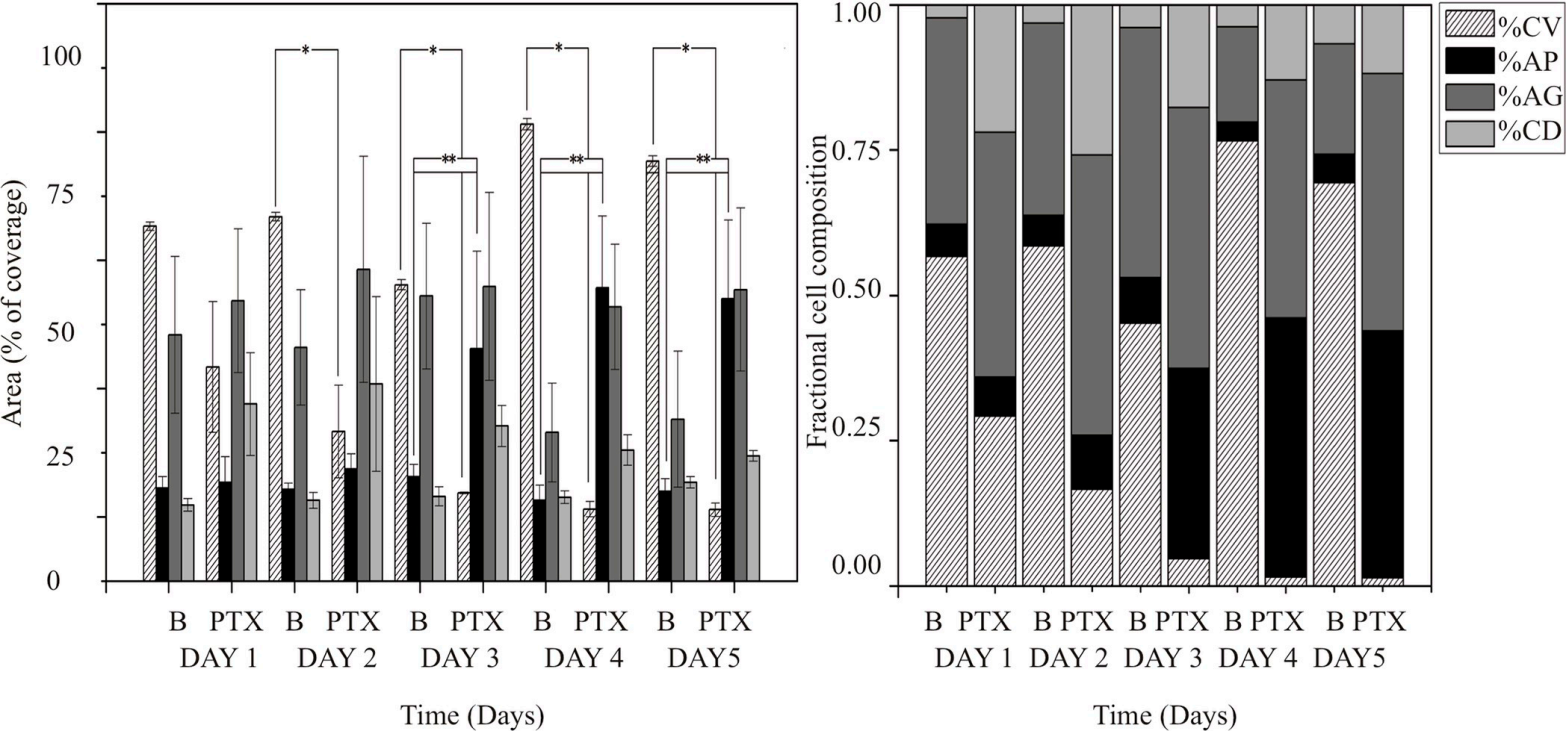
Paclitaxel (PTX) effect compared with control (B) over 5 days at low PTX concentration. Bar graph denotes the value of percentage of the area covered by cells for each of the studied cell death modes. Left, the apoptosis process (**AP**) increased significantly in comparison to the control, and the area coved by viable cells (**CV**) decreased significantly with respect to the control. Right, stacked bar graph shows the value of the results in cell fraction composition per day for a period of five days. (**) and (*) indicates significant difference between days among subgroups (p < 0.05).

The efficacy of paclitaxel was tested at very low concentrations over a period of five days. We observed that this drug had a great influence on **CV** (Fig 6), causing both a decrease in the area of **CV** and an increase in the cells undergoing **AP** with respect to the controls (Fig 6). These results are in agreement with Weigel *et al*. [36], who demonstrated that paclitaxel induces apoptosis in lung cancer cell lines. As an antimicrotubule agent, paclitaxel arrests the G_2_/M-phase transition, interferes with several signal transduction pathways, and induces apoptosis through the stabilization of microtubules [37].

The area of cells with **AG** and **CD** remained almost constant over this same time period.

Metronomic treatment (LDM) with PTX was found to lead to a higher percentage of AP cells and a considerable decrease in the overall percentage of live cells. LDM therapy has been widely reported in recent years in clinical trials and in *in vitro* studies using PTX at very low concentrations [15,38,39]. PTX in LDM (Fig 6 right) was found to induce **AP** and to inhibit cell growth, suggesting that it induces mitotic cell death by disrupting microtubule dynamics and activating the spindle-assembly checkpoint (SAC) [40].

In addition, one of the proapoptotic pathways evaluated was **AG**. Without drug treatment we observed that the percentage of autophagous cells did not decrease over the period of several days studied, but rather remained constant. Apparently, this mechanism can provide extra energy and resources to the cancer cells, allowing their survival in chambered coverslips. The percentage of AG cells remained constant under drug treatment in our experiments. These results are in agreement with the fact that cancer cell exposure to chemotherapy can significantly impact the induction of proapoptotic pathways [41].

## Conclusions

In summary, it was possible to characterize the growth of breast cancer cells within cell chambers and to label them following different treatments. PTX with LDM was found to be the best treatment to induce apoptosis in these cells in chambered coverslips. Furthermore, the **AG** pathway showed an unexpected mechanism for these cells to be able to adapt to growth in the chambered coverslips as well as to the drug treatment.

This method facilitates cell image acquisition in the same spatial position over time which enables to observe cell susceptibility to drugs faster than standard procedures as the developed algorithm allows to perform a reproducible analysis.

The methodology described in this study enabled the quantification of live, dead, autophagous, and apoptotic cells before and after metronomic treatment by live-cell image acquisition and analysis. Two different treatments for breast cancer cells were compared. Paclitaxel in combination with doxorubicin and low doses of paclitaxel. The metronomic paclitaxel treatment (LDM) reported a considerable decrease in the overall percentage of live cells and an increase in the cells undergoing **AP** with respect to the controls. Of note, this new system is a combination of biological, computer science and microfluidics research fields which allowed the quantification of different cell death modes, in particular to determine the percentage of cells that underwent autophagy.

## Supporting information

S1 Table Obtained data represented in Figs 3–6. From analysis using weka segmentation from around 7500 pictures with different stainings Live/Dead Cell Imaging Kit, Autophagy Cell Imaging Kit, Caspase-3 and -7 Cell Imaging Kit, and Propidium iodide. Obtained data illustrated in Fig 3. A) Obtained surface area percentages for cell viability (CV), cell death (CD), cells in apoptosis and cells in autophagy (AG). B) Data from which stacked bar graph illustrating cell fraction composition per day over the five-day period was generated. Obtained data illustrated in Fig 4. C) Data for cell viability (CV), cell death (CD), cells in apoptosis and cells in autophagy (AG) for control and doxorubicin treatment conditions. D) Obtained data to produce stacked bar graph shows the value of the results in cell fraction composition. Obtained data shown in Fig 5. E) Data for cell viability (CV), cell death (CD), cells in apoptosis and cells in autophagy (AG) for control and 4-hour exposure to doxorubicin and a combination of paclitaxel and doxorubicin per day. F) Obtained data to produce stacked bar graph shows the value of the results in cell fraction composition. Obtained data presented in Fig 6. G) Percentage value of the area covered by cells for each of the studied cell death modes for control and paclitaxel treatment conditions. H) value of the results in cell fraction composition per day for a period of five days.
(PDF)
