# Supplementary material for: Metronomic doses and drug schematic combination response tested within chambered coverslips for the treatment of breast cancer cells (JIMT-1)
Source: PLoS One. 2022 Sep 29;17(9):e0274911. doi: 10.1371/journal.pone.0274911 (PMC9522273; doi:10.1371/journal.pone.0274911)
Supplement: S1 Table — From analysis using weka segmentation from around 7500 pictures with different stainings Live/Dead Cell Imaging Kit, Autophagy Cell Imaging Kit, Caspase-3 and -7 Cell Imaging Kit, and Propidium iodide. Obtained data illustrated in Fig 3. A) Obtained surface area percentages for cell viability (CV), cell death (CD), cells in apoptosis and cells in autophagy (AG). B) Data from which stacked bar graph illustrating cell fraction composition per day over the five-day period was generated. Obtained data illustrated in Fig 4. C) Data for cell viability (CV), cell death (CD), cells in apoptosis and cells in autophagy (AG) for control and doxorubicin treatment conditions. D) Obtained data to produce stacked bar graph shows the value of the results in cell fraction composition. Obtained data shown in Fig 5. E) Data for cell viability (CV), cell death (CD), cells in apoptosis and cells in autophagy (AG) for control and 4-hour exposure to doxorubicin and a combination of paclitaxel and doxorubicin per day. F) Obtained data to produce stacked bar graph shows the value of the results in cell fraction composition. Obtained data presented in Fig 6. G) Percentage value of the area covered by cells for each of the studied cell death modes for control and paclitaxel treatment conditions. H) value of the results in cell fraction composition per day for a period of five days. (PDF) [file pone.0274911.s001.pdf]

Supporting information:

S1 Table. Obtained data represented in Figures 3-6. From analysis using weka segmentation from around 7500 pictures with different stainings Live/Dead Cell Imaging Kit, Autophagy Cell Imaging Kit, Caspase-3 and -7 Cell Imaging Kit, and Propidium iodide.

Obtained data illustrated in Figure 3. A) Obtained surface area percentages for cell viability (CV), cell death (CD), cells in apoptosis and cells in autophagy (AG). B) Data from which stacked bar graph illustrating cell fraction composition per day over the five-day period was generated.

Obtained data illustrated in Figure 4. C) Data for cell viability (CV), cell death (CD), cells in apoptosis and cells in autophagy (AG) for control and doxorubicin treatment conditions. D) Obtained data to produce stacked bar graph shows the value of the results in cell fraction composition.

Obtained data shown in Figure 5. E) Data for cell viability (CV), cell death (CD), cells in apoptosis and cells in autophagy (AG) for control and 4-hour exposure to doxorubicin and a combination of paclitaxel and doxorubicin per day. F) Obtained data to produce stacked bar graph shows the value of the results in cell fraction composition.

Obtained data presented in Figure 6. G) Percentage value of the area covered by cells for each of the studied cell death modes for control and paclitaxel treatment conditions. H) value of the results in cell fraction composition per day for a period of five days.

A

Time (Days)	Cell Viability (CV) %	Error	Apoptosis area %	Error	Autophagy process %	Error	Cell Death (CD) area %	Error
3.00	45.19	5.00	7.83	4.00	43.02	11.00	3.95	2.00
4.00	76.54	8.00	3.22	1.00	16.41	5.00	3.82	1.00
5.00	79.32	8.00	3.25	2.00	13.78	2.00	3.73	2.00

B

Time (Days)	Cell Viability (CV) %	Apoptosis area %	Autophagy process %	Cell Death (CD) area %
3.00	0.45	0.08	0.43	0.04
4.00	0.77	0.03	0.16	0.04
5.00	0.79	0.03	0.14	0.04

C

Time (4 Hours)	Cell Viability (CV) %	Error	Apoptosis area %	Error	Autophagy process %	Error	Cell Death (CD) area %	Error
Control	47.25	2.94	8.47	2.00	42.79	11.78	1.49	0.69
DOX	17.45	1.00	42.11	4.00	35.20	17.00	5.23	1.00

D

Time (4 Hours)	Cell Viability (CV) %	Apoptosis area %	Autophagy process %	Cell Death (CD) area %
Control	0.47	0.08	0.43	0.01
DOX	0.17	0.42	0.35	0.05

E

Time (24 Hours)	Cell Viability (CV) %	Error	Apoptosis area %	Error	Autophagy process %	Error	Cell Death (CD) area %	Error
Control	47.25	1	2.61	0.5	43.02	11	2.44	0.68
DOX+PTX	30.79	3	4.43	2.5	54.04	5	10.73	2

F				
Time (24 Hours)	Cell Viability (CV) %	Apoptosis area %	Autophagy process %	Cell Death (CD) area %
Control	0.47	0.04	0.43	0.05
DOX+PTX	0.31	0.04	0.54	0.11

G									
Time (Days)		Cell Viability (CV) %	Error	Apoptosis area %	Error	Autophagy process %	Error	Cell Death (CD) area %	Error
Day 1	B	56.63	0.83	5.62	2.23	35.45	15.23	2.3	1.23
Day 1	PTX	29.2	12.7	6.7	5	42.1	14	22	10
Day 2	B	58.47	0.86	5.32	1.23	33	11.25	3.21	1.56
Day 2	PTX	16.6	9	9.3	3	48.2	22	25.9	17
Day 3	B	45.2	0.98	7.83	2.35	43.02	14.2	3.95	1.87
Day 3	PTX	4.65	0.15	32.75	19	44.9	18.3	17.7	4
Day 4	B	76.55	1.12	3.22	2.89	16.41	9.56	3.82	1.23
Day 4	PTX	1.5	1.5	44.6	14	40.9	12.18	13	3
Day 5	B	69.33	1	4.95	2.45	18.99	13.25	6.73	1.11
Day 5	PTX	1.39	1.31	42.45	15.34	44.28	15.9	11.88	1

H					
Time (Days)	Drug	Cell Viability (CV) %	Apoptosis area %	Autophagy process %	Cell Death (CD) area %
Day 1	B	0.57	0.06	0.35	0.02
Day 1	PTX	0.29	0.07	0.42	0.22
Day 2	B	0.58	0.05	0.33	0.03
Day 2	PTX	0.17	0.09	0.48	0.26
Day 3	B	0.45	0.08	0.43	0.04
Day 3	PTX	0.05	0.33	0.45	0.18
Day 4	B	0.77	0.03	0.16	0.04
Day 4	PTX	0.02	0.45	0.41	0.13
Day 5	B	0.69	0.05	0.19	0.07
Day 5	PTX	0.01	0.42	0.44	0.12
